# Slicing and dicing viruses: antiviral RNA interference in mammals

**DOI:** 10.15252/embj.2018100941

**Published:** 2019-03-15

**Authors:** Pierre V Maillard, Annemarthe G van der Veen, Enzo Z Poirier, Caetano Reis e Sousa

**Affiliations:** ^1^ Division of Infection and Immunity University College London London UK; ^2^ Immunobiology Laboratory The Francis Crick Institute London UK

**Keywords:** antiviral immunity, Dicer, double‐stranded RNA, interferons, RNA interference, Immunology, RNA Biology

## Abstract

To protect against the harmful consequences of viral infections, organisms are equipped with sophisticated antiviral mechanisms, including cell‐intrinsic means to restrict viral replication and propagation. Plant and invertebrate cells utilise mostly RNA interference (RNAi), an RNA‐based mechanism, for cell‐intrinsic immunity to viruses while vertebrates rely on the protein‐based interferon (IFN)‐driven innate immune system for the same purpose. The RNAi machinery is conserved in vertebrate cells, yet whether antiviral RNAi is still active in mammals and functionally relevant to mammalian antiviral defence is intensely debated. Here, we discuss cellular and viral factors that impact on antiviral RNAi and the contexts in which this system might be at play in mammalian resistance to viral infection.

## Introduction

Metazoan organisms are constantly exposed to viruses and have evolved diverse mechanisms to combat the invaders. One group of mechanisms operates in a cell‐intrinsic fashion, targeting viral nucleic acids and viral proteins for destruction and/or causing the premature shutdown or demise of infected cells to prevent them from serving as virus producers. Cell‐intrinsic antiviral mechanisms are part of the innate immune system and include RNA interference (RNAi) and the interferon (IFN) system. The two systems operate very differently even though they can both be triggered by virally derived long double‐stranded RNA (dsRNA) or highly base‐paired single‐stranded RNA (ssRNA). DsRNA can derive from the viral genome (in the case of a dsRNA virus) or from annealing of two strands of complementary RNAs, which are generated as RNA virus replication intermediates or DNA virus convergent transcripts. Highly based‐paired ssRNAs are found in hairpins within viral genomes or viral transcripts and are generically referred to as dsRNA, a nomenclature that we retain here even if technically incorrect. Both types of dsRNA are largely absent from uninfected cells and act as hallmarks of viral infection to trigger innate antiviral immune responses.

**Figure 1:**
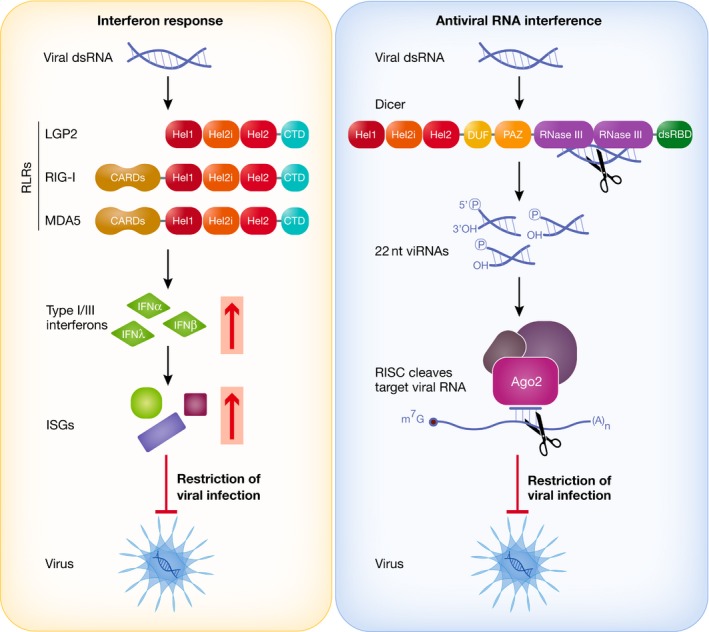
IFN response and antiviral RNAi triggered by viral dsRNA In the cytoplasm of mammalian cells, the RIG‐I‐like receptors (RLRs) RIG‐I and MDA5 detect viral dsRNA and trigger the production of type I interferons, which results in the induction of interferon‐stimulated genes (ISGs) that encode proteins capable of inhibiting viral replication and virus spread. In antiviral RNAi, Dicer cleaves viral dsRNA into viRNAs that are loaded into a RISC complex. As a protein component of this complex, Ago2 degrades viral RNAs with homology to the viRNAs, thereby inhibiting viral replication. RLRs and Dicer share a common DExD/H domain, composed of three helicases (Hel1, Hel2i and Hel2). RIG‐I and MDA5 additionally carry two CARD domains responsible for downstream signalling to MAVS. Dicer possesses two RNase III domains involved in dsRNA dicing.

In RNAi, long dsRNA is cleaved by the type III endoribonuclease Dicer into small interfering RNA (siRNAs) (Bernstein *et al,* 2001), RNA duplexes of 21–24 nucleotides (nts) in length, with 3′ 2‐nt overhangs and a 5′ mono‐phosphate and a 3′ hydroxyl group on both strands (Fig 1) (Hamilton & Baulcombe, 1999; Zamore *et al,* 2000; Elbashir *et al,* 2001b,a). One strand of each siRNA duplex is bound by an Argonaute (Ago) protein, which, together with accessory proteins, forms the RNA‐induced silencing complex (RISC) and mediates the endonucleolytic cleavage (“slicing”) of complementary target RNAs (Hammond *et al,* 2000; MacRae *et al,* 2008). Of the four Ago proteins encoded by the mammalian genome, only Ago2 has catalytic activity and is essential for target slicing and RNAi (Liu *et al,* 2004; Meister *et al,* 2004; Swarts *et al,* 2014; Sheu‐Gruttadauria & MacRae, 2017). However, all four Ago proteins are involved in an RNAi‐related process, the microRNA (miRNA)‐mediated gene silencing pathway, which does not involve slicing but translation inhibition and/or mRNA degradation (Bartel, 2009; Jonas & Izaurralde, 2015). Notably, Dicer is also involved in miRNA biogenesis. Vertebrates and nematodes possess a single Dicer that generates both siRNA and miRNAs while most invertebrates express two Dicer proteins. For example, in *Drosophila melanogaster*, dmDcr‐1 is dedicated to the miRNA pathway while dmDcr‐2 performs antiviral RNAi (Lee *et al,* 2004).

Three observations indicate that RNAi acts as the major antiviral mechanism of plants and invertebrates (Ding & Voinnet, 2007; Kemp & Imler, 2009; Ding, 2010; Sarkies & Miska, 2013; tenOever, 2016). First, viral infections in these organisms lead to the accumulation of Dicer‐dependent virus‐derived siRNAs (viRNAs) that originate from dsRNA viral replication intermediates and/or RNA hairpins and are homologous to viral RNA sequences (Yoo *et al,* 2004; Molnar *et al,* 2005; Galiana‐Arnoux *et al,* 2006; Ho *et al,* 2006; van Rij *et al,* 2006; Wang *et al,* 2006a; Aliyari *et al,* 2008; Félix *et al,* 2011). Second, inactivation of key components of the RNAi pathway results in an increase in viral load in infected cells (Mourrain *et al,* 2000; Dalmay *et al,* 2001; Li *et al,* 2002; Lu *et al,* 2005; Schott *et al,* 2005; Wilkins *et al,* 2005; Deleris *et al,* 2006; Galiana‐Arnoux *et al,* 2006; Wang *et al,* 2006a; Félix *et al,* 2011). Third, many plant and insect viruses encode viral suppressors of RNAi (VSRs) that interfere with distinct steps of the RNAi pathway, demonstrating the selection pressure imposed by this antiviral system (Pumplin & Voinnet, 2013; Bronkhorst & van Rij, 2014; Csorba *et al,* 2015).

In contrast, in chordate cells, including mouse and human cells, dsRNA and other nucleic acids associated with viral infection trigger cytosolic innate immune pathways that induce the production of type I IFNs (mainly IFNα and IFNβ) and type III IFNs (IFNλ) (Goubau *et al,* 2013; Schneider *et al,* 2014; Wu & Chen, 2014; Schlee & Hartmann, 2016) (Fig 1). These key antiviral cytokines are then secreted and act in an autocrine and paracrine manner by binding to their cognate receptors, i.e. the ubiquitously expressed IFNα/β receptor (IFNAR) and the epithelial cell type‐restricted type III IFN receptor (IL‐28R), which signal to induce hundreds of interferon‐stimulated genes (ISGs) (Schneider *et al,* 2014). The proteins encoded by these ISGs limit viral replication directly (Schoggins *et al,* 2011) and serve to enhance adaptive immune responses to the virus (de Veer *et al,* 2001; Iwasaki & Medzhitov, 2010). For example, the dsRNA‐dependent protein kinase R (PKR) is activated by cytosolic dsRNA and phosphorylates and inactivates the eukaryotic translation initiation factor 2 α (eIF2α), resulting in translational arrest and thwarting the production of both viral and host cell proteins (Pindel & Sadler, 2011). This can ultimately lead to the demise of the infected cell, further undermining the ability of the virus to propagate.

Virally derived long dsRNA or highly based‐paired RNA is detected in the cytosol of mammalian cells by RIG‐I like receptors (RLRs), which include RIG‐I (retinoic acid‐inducible gene I), MDA5 (melanoma differentiation factor 5) and LGP2 (laboratory of genetics and physiology 2) (Fig 1). RIG‐I recognises based‐paired ds or ssRNA with a di‐ or triphosphate (5′PP/5′PPP) at its 5′ extremity (Hornung *et al,* 2006; Pichlmair *et al,* 2006; Schlee *et al,* 2009; Schmidt *et al,* 2009; Goubau *et al,* 2014), such as found in the genomes of influenza virus, Sendai virus and reovirus (Baum *et al,* 2010; Rehwinkel *et al,* 2010; Weber *et al,* 2013; Goubau *et al,* 2014). MDA5 triggers comprise long dsRNAs that accumulate during infection with certain viruses such as picornaviruses and reovirus (Gitlin *et al,* 2006; Kato *et al,* 2006; Weber *et al,* 2006; Pichlmair *et al,* 2009; Feng *et al,* 2012). RIG‐I and MDA5 contain two tandem N‐terminal CARDs (caspase activation and recruitment domains) that mediate downstream signalling via the adaptor protein MAVS (mitochondrial antiviral signalling protein), leading to activation of the transcription factors IRF3, IRF7 (interferon regulatory factors 3 and 7) and NFκ‐B (nuclear factor kappa‐light‐chain enhancer of activated B cells). These transcription factors drive the expression of type I and type III IFNs and can directly induce some ISGs. LGP2 lacks CARDs and is unable to induce signalling via MAVS. It is thought to act by modulating responses by the other RLRs (Bruns & Horvath, 2014; Bruns *et al,* 2014; Parisien *et al,* 2018).

Thus, plants and invertebrates lack an IFN system and rely on antiviral RNAi to defend against viruses. In contrast, vertebrates have adopted the IFN system for cell‐intrinsic antiviral defence and are thought to have abandoned antiviral RNAi even though they have retained the RNAi machinery and utilise it for miRNA generation and function. Recently, a number of studies have started to question whether the primordial antiviral function of RNAi has truly been abandoned by mammalian cells or whether it can constitute a physiologically relevant antiviral system that complements the IFN pathway. This has become an area of controversy, with some investigators suggesting that RNAi can be a relevant means of cell‐intrinsic restriction to virus infection in mammals while others argue that it is an epiphenomenon with no role in antiviral resistance (Cullen *et al,* 2013; Cullen, 2014; Ding & Voinnet, 2014; tenOever, 2014, 2017; Jeffrey *et al,* 2017). In this review, we address this controversy and summarise current understanding of antiviral RNAi pathways in mammals and reflect on the possible contexts in which it might play a role.

## Cellular determinants of antiviral RNAi

### Detection of dsRNAi in mammalian cells with attenuated IFN responses

To evaluate the possible existence of antiviral RNAi in mammals, it is useful to consider studies that are exempt from virus‐dependent variables such as expression of VSRs. Therefore, we first discuss studies that use synthetic long dsRNA, composed of two perfectly complementary strands, to trigger RNAi, termed here long dsRNA‐mediated RNAi (dsRNAi). This process depends on the successive processing of long dsRNA into a pool of siRNAs and is distinct from RNAi induced experimentally by the introduction of siRNAs (which bypasses the Dicer machinery) (Caplen *et al,* 2001; Elbashir *et al,* 2001a) or of short hairpin RNAs (shRNAs, which resemble the structure of pre‐miRNAs) (Brummelkamp *et al,* 2002; Paddison *et al,* 2002b; Bartel, 2004).

Long dsRNA‐mediated RNAi was first described in *C. elegans* (Fire *et al,* 1998) followed by *Drosophila*,* Trypanosoma brucei*, planarians and plants (Kennerdell & Carthew, 1998; Ngô *et al,* 1998; Waterhouse *et al,* 1998; Sánchez Alvarado & Newmark, 1999). In mammalian cell lines, long dsRNA had either no effect or displayed a non‐sequence‐specific effect, consistent with activation of the IFN system (Caplen *et al,* 2000; Elbashir *et al,* 2001a). Yet, in preimplantation embryos, as well as in oocytes, embryonic stem cells (ESCs) and embryonal carcinoma (EC) cell lines, the introduction of long dsRNA targeting endogenous genes caused a specific reduction in gene expression and induction of phenotypes comparable to those of null mutants, without causing cell death or translational arrest (Svoboda *et al,* 2000; Wianny & Zernicka‐Goetz, 2000; Billy *et al,* 2001; Yang *et al,* 2001; Paddison *et al,* 2002a). The sequence‐specific silencing induced by long dsRNA in oocytes and ESCs/ECs correlated with a relative inability of these cells to produce and/or respond to IFN (Burke *et al,* 1978; Francis & Lehman, 1989; Stein *et al,* 2005; D'Angelo *et al,* 2016; Wu *et al,* 2018), which suggested that dsRNAi might be active in mammalian undifferentiated cells but masked or inhibited by the IFN system in differentiated cells.

### Antagonism between the IFN response and dsRNAi

**Figure 2:**
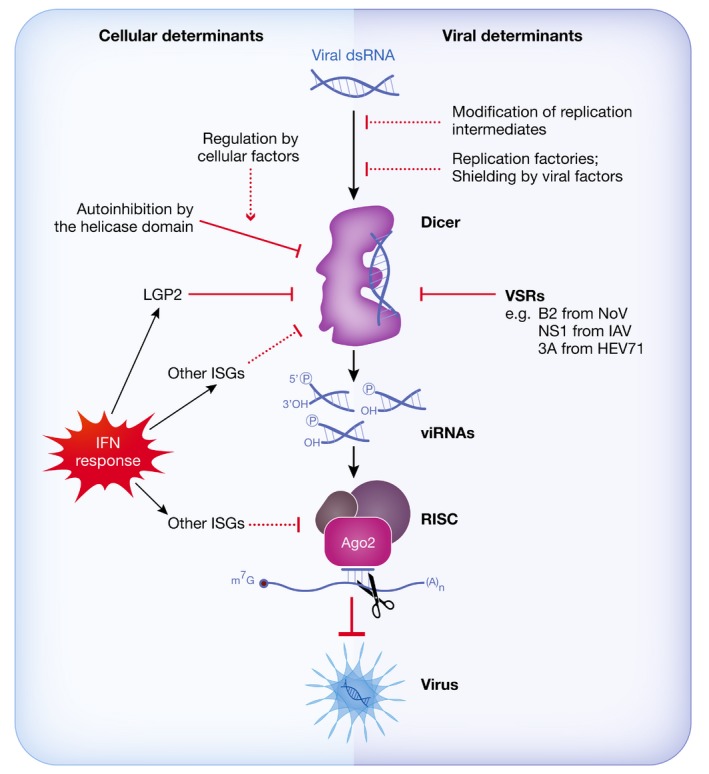
Impact of viral and cellular determinants on antiviral RNAi Recognition and dicing of viral dsRNA by Dicer can be influenced by various viral determinants or cellular factors, as described in main text. Known or putative mechanisms that counteract antiviral RNAi are represented with a plain or dashed red line, respectively. Structure of Dicer is based on Liu *et al* (2018).

Antagonism between the IFN system and dsRNAi was formally tested in somatic cells genetically deficient in MAVS or IFNAR (Maillard *et al,* 2016). In such cells, introduction of dsRNA resulted in Dicer‐dependent accumulation of siRNAs and Ago2‐dependent sequence‐specific gene silencing (Maillard *et al,* 2016). A subsequent study showed that the IFN system actively inhibits dsRNAi at least in part through induction of LGP2, which binds Dicer and inhibits processing of long dsRNA into siRNAs (Van der Veen *et al,* 2018) (Fig 2). In that study (Van der Veen *et al,* 2018), LGP binding to Dicer did not impact the biogenesis of two household miRNAs although LGP2 has also been reported to interact with the Dicer co‐factor TRBP (HIV TAR RNA‐binding protein) and inhibit the processing of a subset of TRBP‐bound miRNAs (Komuro *et al,* 2016; Takahashi *et al,* 2018). Whether LGP2 additionally inhibits dsRNAi via TRBP remains to be addressed.

It is unclear why somatic cells should inhibit dsRNAi during an IFN response. A clue may come from the observation that mammalian cells stably expressing *Drosophila* dcr‐2 to artificially boost dsRNAi have impaired induction of IFN upon treatment with poly(I:C), a dsRNA analog (Girardi *et al,* 2015). Viral infection or treatment with poly(I:C) also induces poly‐ADP‐ribosylation of Ago2 and other RISC components, which inhibits RISC activity and causes a relief in miRNA‐mediated repression of some ISGs (Seo *et al,* 2013). Perhaps, inhibition of Dicer and RISC is essential for effective stimulation of the IFN pathway, in part by preventing loss of dsRNA substrates for RLR activation. Preservation of dsRNA in infected cells may also ensure that the activity of antiviral proteins encoded by ISGs is not compromised. For example, PKR requires dsRNA of > 30 nts to dimerise and become active for translational repression (Husain *et al,* 2012). Dicer‐mediated cleavage of long dsRNA could starve the cell of substrates for PKR activation or, more likely, lead to accumulation of 21–22 nt siRNA duplexes that would “quench” PKR monomers, blocking substrate‐dependent dimerisation.

### Intrinsic inefficiency of mammalian Dicer in processing long dsRNA

DsRNAi in mammalian cells is further influenced by the molecular properties of its central component: Dicer. This large multi‐domain enzyme comprises an N‐terminal DExD/H helicase domain (containing an ATPase site) followed by a small domain of unknown function (DUF283), a Piwi Argonaute Zwille (PAZ) domain, two tandem RNAse III domains and a C‐terminal dsRNA‐binding domain (dsRBD—Fig 1). The PAZ domain binds the 3′ 2nt‐overhangs found at the extremity of dsRNA substrates, while the RNAse III domains each mediate the cleavage of one strand of the RNA duplex. *In vitro* studies revealed that human Dicer (hDcr) processes long dsRNA into siRNAs less efficiently than pre‐miRNA into miRNAs (Ma *et al,* 2008; Chakravarthy *et al,* 2010). Deletion or partial proteolysis of the helicase domain increases rate of dsRNA cleavage, while only modestly affecting the cleavage of pre‐miRNAs (Provost *et al,* 2002; Zhang *et al,* 2002; Ma *et al,* 2008). Similarly, a deletion mutant of hDcr lacking nearly the entire helicase domain displayed an enhanced ability to process endogenously transcribed long dsRNA and long hairpin RNAs into siRNAs and conferred dsRNAi activity to engineered cells (Kennedy *et al,* 2015). Finally, mouse oocytes, in which dsRNAi is active, express a shortened isoform of Dicer (Dicer^O^) that lacks the N‐terminal helicase domain and processes endogenous or ectopically expressed long hairpin RNAs more efficiently (Flemr *et al,* 2013). Together, these data suggest that the helicase domain of Dicer inhibits its catalytic activity for long dsRNA and that its incorporation into the mature enzyme might be regulated by alternative transcription. However, expression of Dicer^O^ has not been detected outside mouse germ cells and expression of truncated Dicer isoforms in humans has been reported only in certain cancer cell lines (Potenza *et al,* 2010; Hinkal *et al,* 2011; Cantini *et al,* 2014). An alternative possibility is that modulation of inhibition by the helicase domain could come about not through alternative transcription but through the activity of Dicer‐associated proteins. Structural studies show that the co‐factors TRBP and PACT (Protein Activator of PKR) induce a conformational change in the Dicer helicase domain (Taylor *et al,* 2013) that could mimic the effect of deletion (Lee *et al,* 2006; Ma *et al,* 2008; Chakravarthy *et al,* 2010; Ota *et al,* 2013). The exact role and cellular context in which TRBP and PACT might modulate the ability of Dicer to process long dsRNA *in vivo* remains to be explored.

## Viral determinants of antiviral RNAi

Antiviral RNAi is distinct from dsRNAi in (i) the origin of the substrate dsRNA (viral RNA vs exogenous sources) and (ii) the RNAs targeted by the RISC (viral RNA vs host cell mRNA). Antiviral RNAi therefore depends on the efficient production of viRNAs from viral dsRNA and the efficient targeting of viral RNA by the RISC machinery.

### Targeting of viral RNA by exogenous small RNAs

Cells transfected with siRNAs or expressing an shRNA targeting viral genomes display sequence‐specific reductions in viral RNA accumulation and virus replication upon challenge with homologous viruses, including human immunodeficiency virus (HIV), hepatitis C virus (HCV), influenza A virus (IAV), West Nile virus (WNV), SARS coronavirus, human papilloma virus and various picornaviruses (Gitlin *et al,* 2002; Jacque *et al,* 2002; Lee *et al,* 2002; Ge *et al,* 2003; Kapadia *et al,* 2003; Konishi *et al,* 2003; Randall *et al,* 2003; Lu *et al,* 2004; Phipps *et al,* 2004; Takigawa *et al,* 2004; Bitko *et al,* 2005; Shi *et al,* 2005; Sim *et al,* 2005; Werk *et al,* 2005; Yuan *et al,* 2005; Kumar *et al,* 2006; Bousarghin *et al,* 2009; Qureshi *et al,* 2018). Similarly, delivery of siRNAs to mice, prior to or concomitant with viral challenge, provides protection against infection with various viruses (Ge *et al,* 2003; Giladi *et al,* 2003; McCaffrey *et al,* 2003; Tompkins *et al,* 2004; Bitko *et al,* 2005; Tan *et al,* 2007; Shah & Schaffer, 2011). RNA viruses engineered to contain perfectly complementary target sites for cellular miRNAs are restricted in cells or tissues expressing the cognate miRNAs (Cawood *et al,* 2009; Perez *et al,* 2009; Kelly *et al,* 2010; Langlois *et al,* 2013). Finally, in IFN‐defective somatic cells, introduction of long dsRNA conferred sequence‐specific protection from viral challenge dependent on the “slicing” activity of Ago2 (Maillard *et al,* 2016). Together, these studies show that, if siRNAs are provided directly (bypassing Dicer processing) or are generated from substrates in the absence of an IFN response (shRNAs or dsRNAs in IFN‐defective cells), RISC can access and target viral RNAs to limit viral accumulation. As such, much of the current debate on the role of antiviral RNAi in mammalian cells ultimately centres on the question of whether, during a viral infection, viRNAs are ever produced in sufficient amounts to engage the latent antiviral activity of RISC and exert an antiviral effect.

### viRNA production and antiviral RNAi in mammalian cells

viRNAs have key features: (i) a discrete length of ~22 nt, (ii) extremities with 3′ 2nt overhangs and (iii) a strand derived from the positive (+)‐sense viral RNA and a complementary strand commonly derived from the negative (−)‐sense viral RNA or, less frequently, from intramolecular base pairing of viral ssRNA. Initial attempts failed to detect viRNAs in mammalian cells infected with viruses (Pfeffer *et al,* 2005; Lin & Cullen, 2007), yet the recent emergence of high‐throughput sequencing has allowed the question to be re‐explored more thoroughly. Deep sequencing of differentiated mammalian cells infected with five mammalian viruses [HCV, dengue virus (DENV), WNV, poliovirus and vesicular stomatitis virus (VSV)] revealed the presence of small RNAs derived from the viral genome (viral small RNAs or vsRNAs) (Parameswaran *et al,* 2010). These vsRNAs, however, did not display size uniformity except in cells carrying a HCV replicon or infected with HCV virions (Parameswaran *et al,* 2010). Additional deep sequencing experiments of human cells infected with a range of viruses [WNV, DENV, Borna disease virus, IAV, Sindbis virus (SINV)] also reported the detection of vsRNAs but not specifically 22 nt long ones (Girardi *et al,* 2013; Backes *et al,* 2014; Bogerd *et al,* 2014). In addition, deep sequencing of RIG‐I‐ and MDA5‐deficient cells infected with SINV, YFV and the picornavirus coxsackie virus B3 did not reveal viRNA accumulation (Schuster *et al,* 2017) [although the inhibition of Dicer activity by LGP2 might have dampened the response (Van der Veen *et al,* 2018)]. Altogether, these experiments argue for limited Dicer‐mediated generation of viRNAs in IFN‐competent differentiated somatic cells.

In contrast, deep sequencing of mESCs infected with the picornavirus encephalomyocarditis virus (EMCV) revealed the accumulation of viral reads with a specific peak at 21‐23 nt and reads that mapped within the first 200‐nt of the EMCV genome and, to a lesser extent, to the 3′end and that, importantly, derived in equal parts from the (+) strand and (−) strand (Maillard *et al,* 2013). The sequences formed perfectly paired duplexes with 3′ 2‐nt overhangs and were produced in a phase pattern indicative of successive cleavage by Dicer. Finally, the duplexes could be shown to associate with Ago2 and require Dicer for their generation, thereby fulfilling all criteria for *bona fide* viRNAs (Maillard *et al,* 2013). Interestingly, production of these viRNAs by mESCs was greatly reduced upon cell differentiation (Maillard *et al,* 2013), in line with the aforementioned studies reporting little viRNA generation in differentiated somatic cells.

Thus, in some IFN‐deficient cells, including ESCs, infection with viruses allows for viRNA production. Can it elicit a protective RNAi‐dependent response? Early work suggested that knockdown of Dicer in Vero cells, an African green monkey cell line that lacks IFN‐α and IFN‐β genes (Diaz *et al,* 1988), causes a modest increase in virus production upon IAV infection (Matskevich & Moelling, 2007). In contrast, later reports found that absence of Dicer in HEK 293T cells and/or in mouse embryonic fibroblasts did not impact the accumulation of flaviviruses (DENV, WNV, YFV), alphaviruses (SINV, Venezuelan equine encephalitis virus [VEEV]), IAV, measles virus, HIV and reovirus (Shapiro *et al,* 2010; Bogerd *et al,* 2014). Similarly, in engineered cells overexpressing an artificial Dicer that lacks the helicase domain, infection with IAV or poliovirus led to low‐level accumulation of viRNAs, which were loaded onto RISC but had little impact on replication (Kennedy *et al,* 2015). Finally, expression of a slicing‐deficient Ago2 mutant in *Ifnar1* ^−/−^ mouse embryonic fibroblasts did not impact infection with Semliki Forest virus (SFV), reovirus or IAV (Maillard *et al,* 2016). The overall message from those studies is that, even in a context permissive for dsRNAi, viRNA production from viral replication intermediates is too weak to inhibit viral infection. Replication of (+)‐sense RNA viruses often occurs within membranous structures (replication factories), whereas RNAs generated during replication of (−)‐sense RNA viruses rapidly associate with nucleocapsid proteins (Conzelmann, 1998; Romero‐Brey & Bartenschlager, 2014). In addition, the 5′ extremities of certain viral genomes (and replication intermediates) can display various modifications, including a 7‐methylguanosine (Cap) structure, a covalently linked protein (e.g. Vpg, viral protein genome‐linked), highly structured regions or 2–3 phosphates (Fig 2). Whether these features prevent efficient access of Dicer to viral RNA is unknown although it is worth remembering they do not prevent antiviral RNAi in insect cells. A key issue might therefore be antagonism of RNAi by VSRs. This will be discussed in the next section.

### VSRs in mammalian viruses

Several proteins encoded by mammalian viruses display VSR activity (1; 2). Expression of influenza virus NS1, vaccinia virus E3L, reovirus σ3 and Nodamura virus (NoV) B2 proteins inhibits RNAi in plants and/or insect cells (Lichner *et al,* 2003; Bucher *et al,* 2004; Delgadillo *et al,* 2004; Li *et al,* 2004). In mammalian cells, a plethora of mammalian virus‐encoded VSRs, including primate foamy virus type 1 (PFV‐1) Tas, NoV B2, HCV core, IAV NS1, HIV Tat, Ebola VP35, VP30 and VP40, CoV N, SARS‐CoV 7a, YFV capsid, DENV NS4B, human enterovirus 71 (HEV 71) 3A and adenovirus virus‐associated RNA I (VA1), can reduce shRNA/siRNA‐mediated knockdown of reporter genes (Table 1) (Lu & Cullen, 2004; Andersson *et al,* 2005; Lecellier *et al,* 2005; Sullivan & Ganem, 2005; Wang *et al,* 2006b; Haasnoot *et al,* 2007; Chen *et al,* 2008; de Vries *et al,* 2009; Karjee *et al,* 2010; Fabozzi *et al,* 2011; Kakumani *et al,* 2013; Cui *et al,* 2015; Samuel *et al,* 2016; Qiu *et al,* 2017). Most viral proteins identified thus far that display VSR activity share the ability to bind dsRNA and mutations that affect their dsRNA‐binding domain block VSR activity, arguing that their principal mode of action is sequestration of dsRNA from Dicer (Table 1). As dsRNA is a potent inducer of the IFN pathway, most of these VSRs also act as IFN antagonists (García‐Sastre, 2017). It is therefore unclear whether these viral proteins specifically evolved to block RNAi or whether their VSR activity is a byproduct of their role as IFN antagonists (Cullen, 2006). However, some VSRs may function through mechanisms other than dsRNA sequestration (Kakumani *et al,* 2013), including binding to components of the RNAi pathway: e.g. Ebola virus VP35 and VP30 proteins interact with Dicer co‐factors TRBP and PACT, while HCV core associates with Dicer (Table 1) (Wang *et al,* 2006b; Chen *et al,* 2008; Fabozzi *et al,* 2011). Whether these interactions contribute to VSR activity is unclear. Finally, adenovirus VA1s are small, highly structured RNAs that inhibit shRNA‐mediated RNAi by acting as decoy substrates for Dicer, RISC and exportin 5 (required for nuclear export of pre‐miRNAs and shRNAs) (Lu & Cullen, 2004; Andersson *et al,* 2005).

**Table 1:** List of mammalian virus‐encoded proteins with VSR activity

Viral genome	Virus name	Virus family	VSR	Properties	Proposed mode of action	References
(+)‐ssRNA	Coronavirus (CoV)	Coronaviridae	N	dsRNA binding	dsRNA sequestration	Cui *et al* (2015)
	Severe acute respiratory syndrome coronavirus (SARS‐CoV)	Coronaviridae	7a	–	–	Karjee *et al* (2010)
	Dengue virus (DENV)	Flaviviridae	NS4B	lack of dsRNA binding	inhibition of Dicer activity	Kakumani *et al* (2013)
	Hepatitis C virus (HCV)	Flaviviridae	capsid	Dicer binding	inhibition of Dicer activity	Wang *et al* (2006b), Chen *et al* (2008)
	Yellow Fever virus (YFV)	Flaviviridae	capsid^a^	dsRNA binding	dsRNA sequestration	Samuel *et al* (2016)
	Human enterovirus 71 (HEV71)	Picornaviridae	3A	dsRNA binding	dsRNA sequestration	Qiu *et al* (2017)
	Human immunodeficiency virus 1 (HIV‐1)	Retroviridae	Tat	dsRNA binding	–	Bennasser *et al* (2005), Triboulet *et al* (2007), Lin and Cullen (2007)^b^, Sanghvi & Steel (2011)^b^
	Primate foamy virus type 1 (PFV‐1)	Retroviridae	Tas^a^	–	–	Lecellier *et al* (2005)
	Nodamura virus (NoV)	Nodaviridae	B2	dsRNA binding	dsRNA sequestration	Sullivan and Ganem (2005), Aliyari *et al* (2008), Li *et al* (2013), Maillard *et al* (2013)
(−)‐ssRNA	Ebolavirus	Filoviridae	VP30	Dicer and TRBP binding	inhibition of Dicer activity	Fabozzi *et al* (2011)
			VP35	PACT, TRBP, dsRNA binding	inhibition of Dicer activity	Haasnoot *et al* (2007), Fabozzi *et al* (2011)
			VP40	–	–	Fabozzi *et al* (2011)
	Marburg virus	Filoviridae	VP35	dsRNA binding	–	Li *et al* (2016)
	Influenza virus	Orthomyxoviridae	NS1	dsRNA binding	dsRNA sequestration	Li *et al* (2004), Bucher *et al* (2004), Delgadillo *et al* (2004), Kok and Jin (2006)^b^, de Vries *et al* (2009), Kennedy *et al* (2015), Benitez *et al* (2015)^b^, Li *et al* (2016), Tsai *et al* (2018)
			NP	–	–	Kennedy *et al* (2015)
	La Crosse virus	Peribunyaviridae	NSs	–	–	Soldan *et al* (2004)
dsRNA	Reovirus	Reoviridae	σ3^a^	dsRNA binding	dsRNA sequestration	Lichner *et al* (2003)
dsDNA	Adenovirus	Adenoviridae	VA I, VA II	Dicer binding	Dicer sequestration by acting as decoy RNAs	Lu and Cullen (2004), Andersson *et al* (2005)
	Vaccinia virus	Poxviridae	E3L	dsRNA binding	dsRNA sequestration	Li *et al* (2004), Haasnoot *et al* (2007)

a VSR activity shown only in non‐mammalian hosts.

b Studies questioning VSR activity.

Despite the evidence that many viral proteins from mammalian viruses can act as VSRs in overexpression (i.e. gain‐of‐function) studies, there are relatively few loss‐of‐function studies that show that they actively suppress mammalian antiviral RNAi defence. Persuasive experiments have been done with NoV, a member of the *Nodavirus* family. Nodaviruses express B2 proteins, which bind long dsRNA and siRNAs *in vitro* and associate with replication intermediates and viRNAs in infected cells (Chao *et al,* 2005; Lu *et al,* 2005; Sullivan & Ganem, 2005; Aliyari *et al,* 2008). B2 proteins act as potent VSRs in insect cells (Wang *et al,* 2006a; Aliyari *et al,* 2008). Notably, B2‐deficient NoV (NoV ΔB2) also replicates less efficiently than parental NoV in mESCs but its accumulation is rescued in mESCs lacking all Ago genes (Maillard *et al,* 2013). In suckling mice, NoV ΔB2 is highly attenuated and induces accumulation of viRNAs (Li *et al,* 2013). viRNAs are also detected, although to a lesser degree, upon infection of somatic cells (BHK‐21) with NoV ΔB2, but not with NoV WT (Li *et al,* 2013). Altogether, these data suggest that the ability of Dicer to process NoV replication intermediates is actively antagonised by the B2 protein.

The NS1 protein from IAV also displays VSR activity when expressed in plants and insect cells (Bucher *et al,* 2004; Delgadillo *et al,* 2004; Li *et al,* 2004). In mammalian cells, NS1 is ineffective against RISC‐loaded siRNAs (Kok & Jin, 2006; Haasnoot *et al,* 2007; de Vries *et al,* 2009; Kennedy *et al,* 2015) but infection of human and African green monkey cells with IAV ΔNS1 but not IAV WT yields readily detectable levels of canonical viRNAs derived from the termini of both strands of the eight viral RNA segments (Li *et al,* 2016; Tsai *et al,* 2018). It has been reported that IAV ΔNS1, and to a lesser extent parental wild‐type IAV, VSV and EMCV, replicates more extensively in mouse embryonic fibroblasts expressing a slicing‐deficient Ago2 mutant (Li *et al,* 2016). However, in other studies, loss of RNAi components did not cause an increase in replication of IAV ΔNS1 (Maillard *et al,* 2016; Tsai *et al,* 2018). Interestingly, IAV engineered to express an shRNA or a miRNA targeting a viral gene or a reporter gene integrated in the viral genome, respectively, was attenuated compared to non‐targeting controls (Benitez *et al,* 2015). This restriction was Dicer‐dependent but independent of NS1 suggesting that, in the context of an infection, NS1′s VSR activity inhibits viRNA production from genome segments but not from short dsRNA hairpins (Benitez *et al,* 2015; Li *et al,* 2016; Tsai *et al,* 2018).

Finally, the HEV71‐encoded protein 3A inhibits shRNA‐mediated silencing in mammalian cells, as well as antiviral RNAi in insect cells, and suppresses Dicer‐mediated biogenesis of siRNAs by binding and sequestering long dsRNA *in vitro* (Qiu *et al,* 2017). A point mutation that inactivates 3A's VSR activity reduces viral replication in somatic cells and in suckling mice. Concomitantly, canonical viRNAs derived from both strands of the 5′terminal region of the HEV71 genome are produced, loaded into RISC and able to silence a reporter bearing complementary sites (Qiu *et al,* 2017). Interestingly, the absence of Dicer increases HEV71 accumulation in infected cells despite the presence of an intact IFN pathway, suggesting that, in this case, antiviral RNAi could function irrespective of the IFN system (Qiu *et al,* 2017).

### Niches for antiviral RNAi?

The generally observed antagonism between the IFN response and dsRNAi suggests that antiviral RNAi may be especially important in cellular niches in which the induction of or the response to IFN is limited. One of those niches might be stem cells. Pluripotent stem cells do not produce IFN upon viral infection or exposure to poly(I:C) and respond poorly to IFN treatment (Chen *et al,* 2010; Hong & Carmichael, 2013; Wang *et al,* 2013; Guo *et al,* 2015; D'Angelo *et al,* 2016). It is unclear why pluripotent stem cells are refractory to IFN but it may have to do with the fact that self‐renewal is incompatible with the anti‐proliferative effects and pro‐apoptotic effects of the cytokines (Hertzog *et al,* 1994; de Veer *et al,* 2001). Furthermore, artificial induction of an IFN response in engineered pluripotent cells compromises differentiation potential (Eggenberger *et al,* 2019). Thus, pluripotent stem cells may be forced to rely on IFN‐independent mechanisms to combat virus infections. These may include the ability to constitutively express some ISGs that confer an efficient and permanent antiviral state (Wu *et al,* 2018). In this scenario, antiviral RNAi would constitute an additional mechanism to protect the integrity and function of tissue stem cells in the face of virus infection and thereby contribute to tissue maintenance, repair and regeneration (Xia *et al,* 2018). Notably, the ability of a virus to infect stem cells might not be needed for its propagation and, therefore, stem cell‐intrinsic antiviral RNAi would benefit the host but not impact on virus transmission.

### Future directions in antiviral RNAi

In line with other facets of immunity, it is likely that antiviral RNAi is highly tuneable and that it operates in conjunction with multiple other mechanisms of defence. Further studies are clearly needed to disentangle the complex web that regulates dsRNAi in mammals and to understand its ability to act as a cell‐intrinsic mechanism of antiviral defence. Open questions include what are the cellular factors regulating the activity of Dicer on long dsRNA? Apart from murine germ cells, are there similar truncated Dicer isoforms expressed in other cell types and/or other species? Which viral proteins act as *bona fide* VSRs in the context of an infection? What are the cell types in which antiviral RNAi is active? Does antiviral RNAi directly impact on viral accumulation upon infection *in vivo*? These and other questions are likely to enliven the debate on the role of RNAi in mammalian defence from virus attack for years to come.

## Conflict of interest

The authors declare that they have no conflict of interest.
